# Too Early to Tell? Balancing Diagnostic Accuracy of Newborn Screening for Propionic Acidemia Versus a Timely Referral

**DOI:** 10.3390/ijns12010001

**Published:** 2025-12-24

**Authors:** Nils W. F. Meijer, Hidde H. Huidekoper, Klaas Koop, Sabine A. Fuchs, M. Rebecca Heiner Fokkema, Charlotte M. A. Lubout, Andrea B. Haijer-Schreuder, Wouter F. Visser, Rendelien K. Verschoof-Puite, Eugènie Dekkers, Annet M. Bosch, Rose E. Maase, Monique G. M. de Sain-van der Velden

**Affiliations:** 1 Department of Genetics, Section Metabolic Diagnostics, University Medical Center Utrecht, 3584 EA Utrecht, The Netherlands; 2 Department of Pediatrics, Center for Lysosomal and Metabolic Diseases, Erasmus MC University Medical Center, 3015 GD Rotterdam, The Netherlands; 3 Laboratory of Metabolic Diseases, Department of Laboratory Medicine, University of Groningen, University Medical Center Groningen, 9700 RB Groningen, The Netherlands; 4 Section of Metabolic Diseases, Beatrix Children’s Hospital, University of Groningen, University Medical Center Groningen, 9700 RB Groningen, The Netherlands; 5 Section of Molecular Metabolism and Nutrition, Laboratory of Pediatrics, University of Groningen, University Medical Center Groningen, 9713 GZ Groningen, The Netherlands; 6 Reference Laboratory for Neonatal Screening, Center for Health Protection, National Institute for Public Health and the Environment (RIVM), 3720 BA Bilthoven, The Netherlands; 7 Department for Vaccine Supply and Prevention Programmes, RIVM Dutch National Institute for Public Health and the Environment, 3720 BA Bilthoven, The Netherlands; 8 Centre for Population Screening, National Institute for Public Health and the Environment, RIVM, 3720 BA Bilthoven, The Netherlands; 9 Amsterdam Gastroenterology Endocrinology and Metabolism, Division of Metabolic Diseases, Department of Pediatrics, Emma Children’s Hospital, Amsterdam UMC, University of Amsterdam, 1105 AZ Amsterdam, The Netherlands

**Keywords:** propionic aciduria, first-tier, expedited referral, European NBS programs, newborn screening

## Abstract

In the Netherlands, the newborn screening (NBS) program includes screening for propionic aciduria (PA) and methylmalonic aciduria (MMA). When initial screening reveals elevated C3 concentrations or abnormal ratios (C3/C2, C3/C16), a second-tier test measuring methylcitric acid (MCA) for PA and methylmalonic acid (MMA^mb^) for MMA is performed. While this two-tier approach reduces false positives effectively, it can delay referral from the NBS program and diagnosis of propionic aciduria. We describe four early-onset PA cases in which the current Dutch screening algorithm negatively impacted clinical outcomes, highlighting the need for expedited referral. We investigated different alternative screening strategies to identify the most effective approach for improving timeliness, while maintaining the high specificity of Dutch PA NBS. This revised approach prioritizes the evaluation of the C3/C2 ratio in first-tier screening. Specifically, samples with a C3/C2 ratio ≥ 0.75 should be referred directly for medical consultation and confirmatory testing. For all other samples with less pronounced biochemical abnormalities, the existing two-tier screening algorithm remains an appropriate NBS protocol. To position our approach internationally, a survey of European NBS programs was conducted to compare screening and referral protocols for PA across the region.

## 1. Introduction

Propionic aciduria (PA) (OMIM #606054) is a rare, inherited metabolic disorder caused by bi-allelic pathogenic variants in the genes encoding one of the two subunits of the propionyl-CoA carboxylase (PCC) enzyme (EC 6.4.1.3). This enzyme is responsible for converting propionyl-coenzyme A (propionyl-CoA) to methylmalonyl-CoA. A deficiency of PCC leads to the accumulation of toxic metabolites, with both acute life-threatening presentations with metabolic acidosis, hyperammonemia, neurological impairment, thermoregulatory instability and multi-organ damage, and as a chronic, progressive disease in which virtually every organ system can be affected. The clinical picture of PA is heterogeneous. In general, PA presents in two forms. Early-onset PA, defined as a clinical presentation < 29 days of life [1], typically presents within the first days to weeks of life with non-specific symptoms such as vomiting, lethargy, hypotonia and feeding difficulties. Laboratory tests will show metabolic acidosis and potentially life-threatening hyperammonemia. Conversely, late-onset PA may develop months or even years after birth with more variable and attenuated symptoms [2]. Upon diagnosis, treatment with a protein-restricted diet and L-carnitine supplementation is initiated to minimize the risk of metabolic decompensation and promote the excretion of accumulating toxic metabolites [3].

Newborn screening (NBS) for PA is not universally implemented, with significant variability across countries, depending on health policies, resources, and healthcare priorities. Also, even though NBS for PA has been shown to reduce mortality, it may not affect long-term outcome [4]. In Europe, 19 of the 48 countries currently include PA in their routine NBS programs [1,5]. The acylcarnitine profile in samples from newborns with PA typically shows an elevated concentration of propionylcarnitine (C3). However, the specificity of C3 is relatively low, as its concentration can be influenced by various factors such as gestational age, infection, parenteral nutrition, prolonged hyperbilirubinemia, (maternal) cobalamin deficiency or genetic methylmalonic acidemias [6]. Consequently, NBS for PA leads to many false positive referrals when based solely on C3 concentrations [7]. As a result, some screening programs have discontinued or chosen not to implement NBS for PA. Other programs have introduced first-tier strategies to reduce the incidence of false positives in NBS for PA, including the use of age-adjusted cut-off values and metabolite ratios such as C3/C2, C3/C16, and C3/methionine to enhance diagnostic accuracy. Additionally, some laboratories have introduced second-tier testing—for example, by measuring 3-hydroxypropionic acid and/or methylcitric acid (MCA) from the original DBS sample, thereby improving the positive predictive value of NBS for PA [8,9,10,11,12,13]. However, the widespread implementation of 3-hydroxypropionic acid and MCA testing is limited by logistical constraints and the requirement for specialized equipment and expertise. Additionally, introducing second-tier testing will delay the referral of patients potentially developing a life-threatening metabolic decompensation.

PA has been included in the Dutch NBS program since October 2019 and was implemented together with Methylmalonic acidemia (MMA). When advising about the PA screening, the Dutch Health Council acknowledged that children with late onset PA would be more likely to benefit from early detection through NBS because of the timing of the heel prick in The Netherlands. Infants with the severe early-onset form were expected to be symptomatic before the availability of NBS results, and for these patients, the health benefit of NBS was therefore considered limited. The Dutch NBS protocol was developed to screen for the target condition late-onset PA. The Dutch primary screening algorithm in the first tier is identical for PA and MMA and relies on C3, C3/C2, and C3/C16 ratios, with MCA and methylmalonic acid (MMA^mb^; mb refers to metabolite) used as second-tier markers, respectively, to differentiate between the two disorders. Whilst second-tier analysis is time-consuming, this was considered acceptable for NBS for late onset PA, the target condition, although this delays referral from the NBS program and the subsequent diagnosis of patients with early-onset PA. Especially for these patients, early identification of PA is critical to initiate treatment before irreversible damage occurs.

In this paper, we present four cases of early-onset PA, detected though the Dutch NBS program, in which the delay in referral from the NBS program caused by the deployment of second-tier testing adversely affected clinical outcomes. This highlights the need for an expedited screening for newborns with early-onset PA to enable timely intervention. To position this in an international context, we conducted a Europe-wide survey using a structured questionnaire to gather detailed information on PA screening algorithms and referral protocols employed in the different European NBS programs. Based on our NBS data, we propose an adjusted screening approach which will allow for the rapid detection of early-onset PA patients without second-tier MCA testing delays. Given the shared first-tier algorithm for PA and MMA, the newly developed protocol was additionally evaluated for its impact on MMA.

## 2. Materials and Method

### 2.1. Case Descriptions

Four Dutch patients with early-onset PA who became symptomatic prior to obtaining NBS results are described in detail, including demographic data, NBS markers and initial clinical presentation and metabolic work-up. Written informed consent for publication was obtained from the parents/legal guardians of all four patients.

### 2.2. Data Source

NBS data from 1 January 2018, to 31 December 2024, were retrospectively obtained from the registry maintained by the Dutch National Institute for Public Health and the Environment, Department for Vaccine Supply and Prevention Programs (RIVM-DVP). Approval for the use of these data was granted by the Data Applications Committee of Praeventis at RIVM-DVP. Each NBS dataset included concentrations of all biomarkers measured using the NeoBase™ 2 kit from Revvity (Turku, Finland). Only data from parents who did not object (before 2023) or provided consent (since 2023) for research use were included. Prior to analysis, all data were pseudonymized.

### 2.3. Data Inclusion

The study population included data from de-identified healthy controls and from samples that proceeded to second-tier confirmatory testing for PA and MMA (a multiplex analysis for both conditions). Datasets derived from neonates referred for conditions other than PA and MMA were not included in the study. Neonates between 3 and 183 days of age were included in the study. The target window for the collection of NBS dried blood spots (DBS) in the Netherlands is between 72 and 168 h (3–7 days), while screening is offered up to the age of 6 months (183 days). Neonates screened prior to the target window were excluded as these may include severely ill children that would limit generalization of the results. Datasets derived from neonates with a birthweight between 500 and 6000 g were included in the study, as were neonates with a gestational age between 161 and 308 days.

### 2.4. Dutch NBS Screening for PA (And MMA)

**Figure 1:**
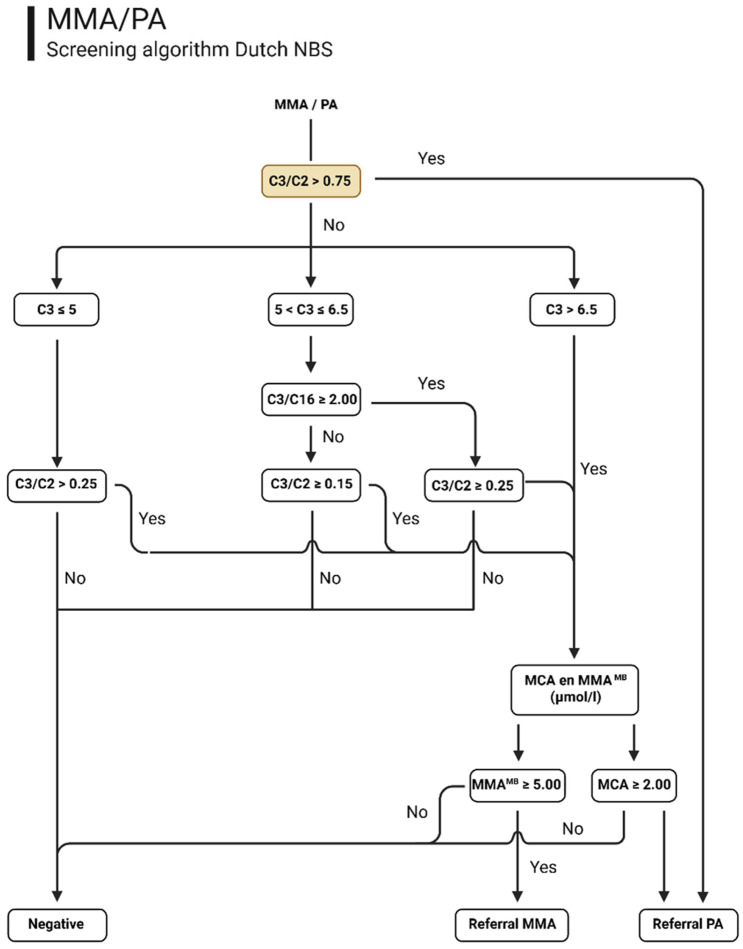
Schematic representation of the current (white) and proposed addition (orange) to the Dutch NBS algorithm. This figure was created with BioRender.com.

The Dutch NBS PA and MMA algorithm consists of two tiers: in the first tier, acylcarnitine profiling using defined cut-off values to evaluate C3, and the C3/C2 and C3/C16 ratios is used. If the first-tier markers are above the predefined nationwide cut-off values, second-tier testing for MCA and MMA is conducted (Figure 1). The first-tier screening is conducted daily at all Dutch NBS laboratories, and the second-tier analysis is centralized at one laboratory and conducted twice a week. The second-tier test is a Liquid Chromatography tandem mass spectrometry-(LC-MS/MS) based method, in which MCA and MMA^MB^ are determined for the PA and methylmalonic aciduria (MMA-OMIM# 251000) screening, respectively. Over the six-year period of screening for PA and MMA, 6072 samples were selected for a second-tier test. Patients were only referred from the NBS program if the second-tier test result was abnormal. If there is insufficient DBS material for second-tier testing, immediate referral is made based on C3 concentration exceeding 10 µmol/L and/or a C3/C2 ratio greater than or equal to 0.35. For milder elevations of the first-tier marker (those equal to or below 10 µmol/L and/or a C3/C2 ratio below 0.35) and in the event of insufficient material for a second tier, a repeat DBS sample is requested. A diagnosis of PA is confirmed through further metabolic investigations of urine and/or plasma, as well as molecular analysis of PCCA and PCCB. Thus far, no false negative cases have been reported for PA (or MMA).

### 2.5. Recognition of Critical Screening Indicators for Urgent Referral

In order to establish one, or more, NBS biomarker(s) that could be used for immediate referral for PA, first-tier marker (i.e., C3, C3/C2 and C3/C16) data from nearly one million newborns with a negative NBS result were used to establish the 99th percentile reference values and were compared with data from the four PA patients described in this study. To further examine the markers, a retrospective reevaluation of all datasets of newborns that were sent for a second-tier MCA/MMAMB test within the Dutch NBS program (from 1 January 2018 to 31 December 2024) was performed using the selection of informative markers, including: C3, C3/C0, C3/C2 and C3/C16. To this end, we determined referral numbers, including those classified as true positive and false positive for PA as well as all datasets belonging to newborns that were characterized as negative based on the second-tier MCA/MMA^MB^ test. Our study aim was to achieve health gain for patients with early-onset PA detected through the Dutch NBS program. Since a multiplex analysis is established for the second tier for PA, in which MMA is also evaluated, we also included the true positive and false positive cases from MMA. However, this was with the sole purpose of understanding the impact of any algorithm changes on the NBS for both PA and MMA and mitigate/avoid any impact on the NBS for MMA. A primary criterion for selection was ensuring that the four early-onset cases would be referred directly based on the new algorithm without adversely affecting the sensitivity and specificity of the PA/MMA screening algorithm. Given the potential relevance of glycine as an additional marker for PA, [2,14] glycine concentrations were retrospectively reviewed in all datasets.

### 2.6. Questionnaire

A structured questionnaire was developed and electronically disseminated to European NBS program coordinators and laboratory directors (*n* = 19) cited as NBS programs which include PA in their NBS program [1] to collect comprehensive information on European practices and experiences related to NBS for PA. The questionnaire included questions on the date of PA NBS implementation, the age at which the heel prick is performed the target condition (early onset, late-onset, or all forms of PA), and enabled detailed descriptions of the PA screening algorithms, including biomarkers and/or marker ratios, and the annual number of samples analyzed in each screening tier employed as appropriate. It also inquired about any additional testing for PA included in the NBS program, the positive predictive value (PPV) of NBS for PA (if known), and questions related to perceived challenges or limitations in PA screening.

## 3. Results

We identified four early-onset PA cases who presented with symptoms before NBS results became available (see details below) out of a total of 11 DBS samples that tested positive for PA. The total screened population consisted of almost 1 million newborns during the study period.

### 3.1. Case Descriptions

#### 3.1.1. Case 1 Early Onset PA

Case 1, the first child of non-consanguineous parents, was born at 40 weeks and 6 days of gestation following an uneventful pregnancy and delivery, with a birth weight of 3380 g. The immediate postnatal period was unremarkable. On day of life (DOL) 3, the parents reported breastfeeding difficulties. On evaluation, the infant appeared alert and clinically well, with a temperature of 36.1 °C and normal vital signs. She was admitted to the hospital, and empiric antibiotic therapy for early-onset sepsis was initiated. The following day, the antibiotics were discontinued due to the absence of clinical and laboratory evidence of infection and persistently low inflammatory markers. Because of continued feeding difficulties, a nasogastric tube was placed. On DOL 6, she started vomiting and developed progressive apathy. By DOL 7, she was encephalopathic, responding only to painful stimuli. Laboratory studies showed a pH of 7.42, pCO_2_ of 4 kPa, bicarbonate of 22.1 (normal 22–29 mmol/L), base excess of −4 mmol/L, and normal glucose, lactate and electrolyte levels. Serum ammonia was markedly elevated at 499 µmol/L. Intravenous glucose infusion (10 mg/kg/min) was started, enteral feeds were stopped, and she was transferred to the pediatric intensive care unit (PICU). Hemodialysis was promptly initiated, with a pre-dialysis ammonia concentration of 587 µmol/L. At this point, we did not start scavengers or carbaglumic acid. After one day of dialysis, ammonia decreased to 82 µmol/L, at which point dialysis was discontinued and she was weaned from mechanical ventilation. A low-protein diet was initiated, along with carnitine, arginine, and ammonia-scavenging agents (sodium phenylbutyrate and sodium benzoate), later supplemented with carglumic acid. Plasma testing on DOL 7 revealed a markedly elevated C3-carnitine level (9.78 µmol/L; normal < 0.50), low C0 carnitine (2.26 µmol/L; normal > 9.62), and a normal plasma amino acid profile (including glycine, glutamine, and glutamic acid). Urinary organic acid analysis supported the diagnosis of PA, corroborated by elevated MCA in plasma (110.35 µmol/L; normal < 0.83). By DOL 8, she was clinically alert. Oral feeding was started. She maintained normal ammonia levels and acid-base status. Brain MRI showed diffusion restriction in the basal ganglia and subthalamic nuclei. The NBS was taken at DOL 5 and the positive result from the NBS was received on DOL 11. Genetic analysis revealed a homozygous PCCA variant: c.923dupT, p.(Leu308Phefs*35). At 7 months of age, she experienced a single seizure, now well-controlled with levetiracetam. Since then, there have been no further seizures or metabolic decompensations. At 9 months, she showed good developmental progress: she sat unsupported, crawled, babbled and was highly responsive, with growth parameters within the normal range. To prevent further decompensations, she recently underwent a liver transplantation just before the age of 1 year. Since, she is more energetic and is doing very well.

#### 3.1.2. Case 2 Early Onset PA

The patient, the second child of non-consanguineous parents, was born at term (40 weeks and 0 days of gestation) following an uneventful pregnancy and delivery. The birth weight was 3740 g. The immediate postnatal period was unremarkable. However, from DOL 1 onwards, the infant experienced feeding difficulties and a 9.5% weight loss. On DOL 7, she was admitted to a general hospital, where tube feeding was initiated and well tolerated. Despite being alert, her energy level remained insufficient for oral feeding. A NBS heel prick was performed on DOL 5, with analysis conducted on DOL 6. The results were inconclusive for OCTN2 deficiency because of a low C0 (3.4 umol/L), and the hospital where the patient was admitted was asked for a second NBS bloodspot. The result of an elevated C3-carnitine level in this first DBS was initially not communicated, as is routine NBS protocol, and the sample was forwarded for second-tier analyses.

The general hospital contacted the metabolic pediatrician due to the inconclusive OCTN2 result. At that point, the low carnitine concentration was not considered suspicious, and a decision was made to await the results of a second NBS. Based on the clinical presentation of lack of energy for drinking, including mention of a large anterior fontanel, it was decided to send plasma and urine for peroxisomal screening.

The second bloodspot was collected on DOL 9 and confirmed the low C0 (2.6 umol/L) from the first blood spot. Of note, the conduction and processing of a second heel prick for OCTN2 does not result in a delay for the PA results. On DOL 10, the pediatrician was informed of an abnormal NBS result for PA (based on the second tier in the first bloodspot). Routine laboratory investigations were advised, revealing an elevated ammonia level of 270 µmol/L, along with a pH of 7.42. Intravenous glucose infusion at 10 mg/kg/min was started, along with sodium benzoate and carnitine supplementation. Enteral feeding was stopped, and the patient was transferred to the pediatric intensive care unit (PICU) for further management. Clinically, the patient soon showed stabilization with an ammonia of 114 umol/L at admission. Metabolic workup showed a biochemical profile consistent with PA with an elevated plasma C3 of 55.3 umol/L (under carnitine supplementation), high plasma glycine (497 umol/L) and urine organic acids revealing a high excretion of 3-hydroxypropionic acid and methylcitric acid in addition to elevated fumaric acid and malic acid. Later, the diagnosis of PA was confirmed both enzymatically (Propionyl-CoA carboxylase below detection) and genetically (homozygous *PCCA* variant: c.923dupT, p.(Leu308Phefs*35)).

Sodiumbenzoate was stopped the next day. Thereafter, she had a stable course and started drinking herself. The patient was started on a low-protein diet and carglumic acid supplementation, and carnitine was continued. Cranial ultrasound at DOL18 showed a small area of increased echogenicity in the thalamic region suspected for focal edema. Because of a good clinical state, she was discharged on DOL18.

There were some hospitalizations because of intercurrent illness without clinical or biochemical signs of metabolic decompensation. Growth and development were both in the normal range; however, from age 8 months, nasogastric tube feeding was started because of feeding problems. The MRI at age 3 months was normal. She showed a variable prolonged QTc.

At the age of 12 months, she received a liver transplantation (living related non carrier donor, uncomplicated procedure). At age 18 months, she is in a good clinical condition with normal development. She eats solids herself, needing the nasogastric tube only for sufficient fluid intake.

#### 3.1.3. Case 3 Early Onset PA

Case 3 is the first child of non-consanguineous parents, was born at term (41 weeks and 3 days of gestation) following an uneventful pregnancy and delivery. The birth weight was 3275 g, and the immediate postnatal period was unremarkable. From DOL 3, the infant developed feeding difficulties and was admitted to a local hospital on DOL 4 due to fluctuating consciousness and hypothermia (35.2 °C). Laboratory investigations revealed a severe metabolic acidosis (pH 7.0, HCO_3^−^_ 3.0 mmol/L, pCO_2_ 2.1 kPa) and marked hyperammonemia (667 µmol/L). Intravenous glucose infusion at 10 mg/kg/min was initiated, and enteral feeding was discontinued. The same day, the patient was transferred to the PICU where hemodialysis and ammonia scavenger therapy were promptly initiated. After two days of dialysis, plasma ammonia levels had decreased to 33 µmol/L, at which point dialysis was stopped and the patient was weaned from mechanical ventilation. Metabolic work-up on the day of admission (DOL 4) revealed elevated C3 carnitine (8.14 µmol/L), low C2 carnitine (1.7 µmol/L) and C0 carnitine (12 µmol/L), with normal methylmalonylcarnitine (C4DC). Plasma amino acids showed slightly elevated glycine (465 µmol/L; normal < 445) and lysine (382 µmol/L) and reduced threonine and alanine. The findings were consistent with PA, confirmed by the detection of a homozygous *PCCA* variant: c.923dupT; p.(Leu308Phe), and in line with NBS results. NBS heel prick was performed on DOL 5. Referral for PA was based on the second tier on DOL 12.

The patient was started on a low-protein diet with carnitine and carglumic acid supplementation. She was discharged on DOL 15. During the first year of life, feeding problems remained, and motor development was delayed. At the age of 9 months, she received a living non-related liver transplant. At this moment, one year after transplantation, no metabolic decompensations have occurred since, and her motor development has improved tremendously.

#### 3.1.4. Case 4 Early Onset PA

Case 4 is a girl, born at 38 weeks and 4 days of gestation after an uneventful pregnancy and delivery, with a birth weight of 2970 g. She was the second child of consanguineous parents with a healthy sister of almost 2 years old. She started breast feeding well but initially had difficulty maintaining adequate body temperature despite external warming. She was discharged at DOL 2. Due to initially insufficient breast milk, additional bottle feeding was started. From DOL 3 onward, breast milk production was sufficient, but the patient became somnolent, fed poorly and lost 14% of her birth weight. She was admitted to a regional hospital on DOL 4, where gastric feeding was started. She developed a progressive metabolic acidosis and mild hyperammonemia (153 µmol/L) on DOL 6, which was first attributed to a difficult blood draw. NBS was performed on DOL 6. The patient developed progressive encephalopathy on DOL 7; ammonia analysis was repeated and was found to be 264 µmol/L. Feeding was stopped immediately, an IV glucose drip of 10 mg/kg/min was started and the patient was transferred to a Pediatric Intensive Care Unit (PICU), where carnitine, sodium benzoate, carglumic acid, hydroxycobalamin and biotin were initiated. An IV lipid emulsion was added for caloric support. Ammonia at admission was 1003 µmol/L, which prompted the need for continuous veno-venous hemodiafiltration (CVVHDF). Ammonia had decreased to 850 µmol/L with medical treatment prior to starting dialysis and normalized within 12 h after CVVHDF initiation and CVVHDF was stopped. Metabolic work-up on DOL 8 revealed elevated C3 carnitine (6.7 µmol/L), low C2 carnitine (1.2 µmol/L) and C0 carnitine (5 µmol/L), with normal methylmalonylcarnitine (C4DC). Plasma amino acids showed elevated glycine (575 µmol/L; normal <445) and reduced glutamine, threonine, alanine, and methionine. The findings were consistent with PA, confirmed by the detection of a homozygous *PCCA* variant: c.1409T>G; p.(Leu470Arg). The patient was started on a protein-restricted diet and continued carnitine supplementation. On DOL 9, she had a single seizure treated with phenobarbital, but otherwise, her admission was uneventful, without any other neurological sequelae. The positive result from the NBS was received on DOL 13. The brain MRI at DOL 17 was normal. After the parents were trained in providing gastric feeding, she could be discharged at DOL 23. Due to multiple metabolic decompensations during the first two years of life, she underwent orthotopic liver transplantation at age 2 years and 11 months. The pre-transplant brain MRI (age 2.9 years) showed mild cortical and cerebellar atrophy, normal myelination, and no basal ganglia abnormalities. Since then, she has remained metabolically stable, with improved energy, appetite, and psychomotor development. At age 5.1 years, her developmental age was estimated at 13 months (Bayley-III-NL). Growth has been adequate.

### 3.2. Comparison of Decision Limits to Detect PA

All first-tier markers were able to distinguish the four early-onset PA cases from healthy newborns (Table 1). Of the five metabolites / metabolite ratios selected for further analysis, C3/C2, C3/C16, and the combination of C3 with C3/C0 all led to the immediate referral of all four early-onset cases. However, using C3/C16 alone as a direct referral criterion resulted in 808 false positives and 5 previously positive MMA cases (Table 2), making it unsuitable as a standalone marker. Referral based on C3 ≥ 20 µmol/L or C3/C0 ≥ 0.5 resulted in the direct referral of 7 and 6 PA patients, respectively (including one and three of the four early-onset PA patients, respectively). A combined criterion of either C3 ≥ 20 µmol/L or C3/C0 ≥ 0.5 successfully identified 10 PA patients, including the four early-onset cases. However, this approach also resulted in the referral of 3 previously NBS-positive MMA cases and 2 previously NBS-negative cases. The most informative single marker proved to be C3/C2 ≥ 0.75, which correctly triggered referral for 10 PA patients, including the four early-onset cases, while leading to the referral of only one true positive MMA case (Table 2). The PA patient with milder biochemical abnormalities in the first tier, most likely associated with late onset PA, would not have been identified through initial direct referral but through second-tier testing, which ensures that cases are not missed. Glycine concentrations in PA patients ranged from approximately 232 to 706 µmol/L, while no patients surpassed the 99th percentile (763 µmol/L) observed in healthy newborns (Supplementary Table S1), suggesting that glycine is not an informative NBS marker for PA.

Based on these findings, we propose an adaptation of the current algorithm (Figure 1). The proposed algorithm includes three potential screening outcomes: screen-negative results requiring no further action; immediate referral based on an increased C3/C2 ratio; and first-tier positive with less pronounced first-tier abnormalities that necessitate second-tier testing.

### 3.3. NBS Programs for PA in Surveyed Countries

**Figure 2:**
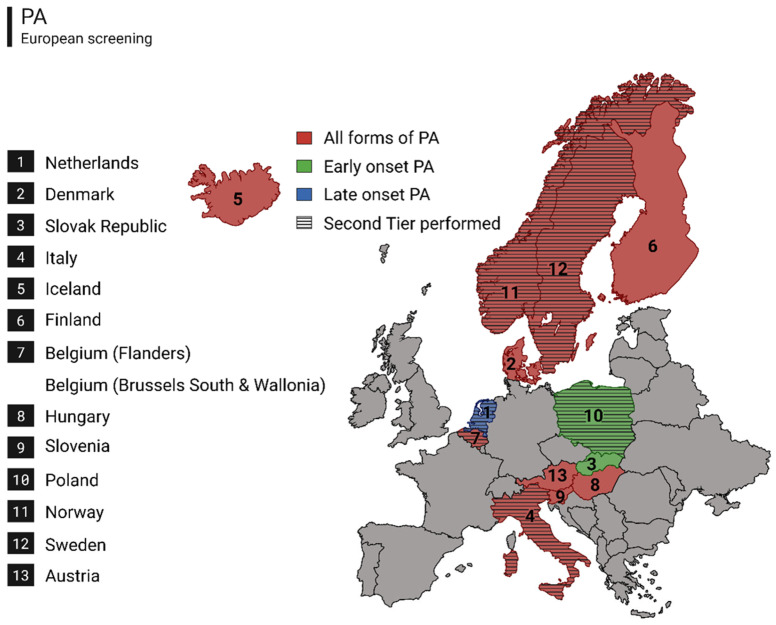
Map of Europe indicating 13 countries (including 14 programs) that screen for PA; colors indicate the form of PA on which each country screens with all forms of PA (red), early onset PA (green) and late onset PA (blue). Countries that utilize both a first and a second tier are hatched. This figure was created with BioRender.com.

Of the 19 NBS programs that we approached, 13 completed the questionnaire (Figure 2 and Table 3). One program (Serbia) informed us that PA is not included in their NBS program. The European PA screening programs evaluated in this work, including The Netherlands, were implemented between 2002 and 2019 in a relatively even distribution (8 prior to 2010, 6 between 2012 and 2019). The Netherlands is unique in its target disease definition of ‘Late-onset PA’; the majority of programs screen for all forms of PA (*n* = 11) and a minority of programs specify early-onset PA as their target condition (*n* = 2). Most programs obtain the heel prick within a timeframe of 48–72 h of age (*n* = 7), one program samples as late as 120 h of age and two programs sample at 36 h of life. For the first-tier screening algorithm, all respondents reported using MS/MS-based analysis of acylcarnitines. In the first tier, all programs utilize the C3/C2 ratio and all but one program utilize C3, with cut-off values varying across programs. The program that does not utilize C3, employs two ratio’s in the first tier (C3/C2 and C4/C3) together with an extensive second tier (methionine, MMA^MB^, MCA and homocysteine). The combinations of markers and cut-offs of screening markers for PA vary internationally.

The annual number of first-tier samples analyzed ranged from 4500 to 250,000 samples, reflecting differences in program scale and population size. Second-tier testing was reported by almost half of respondents (*n* = 4) employing MCA or other related markers in DBS for PA, while two programs focused solely on MMA as the second tier. The interval between first- and second-tier analysis ranged from several hours to one week, depending on laboratory workflow and sample logistics. In total, 4 of 13 respondents made a distinction between urgent and nonurgent referrals, using a different cut-off value for urgent referrals or results obtained on Friday or during holidays. The use of postanalytical tools applied to first-tier screening data with the Collaborative Laboratory Integrated Reports (CLIR) to reduce false-positive cases was reported by three respondents. Challenges and limitations reported by respondents included false positives, the biochemical overlap of NBS biomarkers with other disorders, the rarity of late-onset forms and logistical barriers to (timely) second-tier testing.

## 4. Discussion

In this paper, we present four cases of severe neonatal metabolic decompensation due to PA, which all became symptomatic before PA NBS results were available. Early diagnosis is critical for PA, which frequently presents with acute metabolic crises that can cause irreversible neurological damage if not promptly managed [15]. While some argue that the benefit of NBS is limited because symptoms often appear before results are returned [16], our experience suggests that a modest acceleration in the current screening algorithm may enable earlier, targeted intervention and shorten the initial metabolic decompensation, which can impact the neurological outcome. This view is supported by Heringer et al., who showed that when NBS results are available on DOL 8, 63% of individuals with PA are still asymptomatic and may benefit from early intervention [17]. The Health Council of the Netherlands advised the Ministry of Health, Welfare and Sport to include PA in the NBS panel primarily to benefit individuals with late-onset PA phenotypes, in which metabolic decompensation may be completely prevented [17]. Although in case 3 the patient was already diagnosed before the heelprick was performed, the clinical course observed in these four patients with early-onset PA underscores that rapid availability of NBS data could also benefit early-onset PA cases, as this will shorten the time to initiation of effective treatment aimed at reducing toxic metabolites that may cause irreversible brain damage. Early indicators also play a key role in guiding differential diagnosis, helping clinicians distinguish PA from other more common causes of neonatal distress like sepsis or differentiation from other intoxication-type metabolic conditions such as urea cycle disorders. These conditions may require different treatment strategies: urea cycle defects necessitate nitrogen scavenging, while organic acidemias like PA involve both toxic organic acid accumulation and hyperammonemia, and require specific therapies aimed at correcting those metabolic disturbances. Proposed PA management [3] commends prompt initiation of protein restriction, intravenous fluids, carnitine supplementation and treatment for hyperammonemia (which can include carglumic acid, sodiumbenzoate or even hemodialysis if ammonia is markedly elevated)—treatments that are most effective when started early during metabolic decompensation. Therefore, accelerating the availability of NBS results is crucial in supporting urgent clinical decision-making during emergencies and shortening the duration of metabolic encephalopathy.

There is considerable variation across Europe for NBS of PA. Differences exist in inclusion of PA in national screening programs and, when included, in the use of different biomarkers and follow-up protocols. To better understand and map these discrepancies, we conducted a survey among 19 European NBS centers, of which 13 responded. The results highlight substantial heterogeneity in screening practices, underlining the need for greater harmonization to ensure equal chances of early detection and treatment for all affected newborns across Europe. The primary challenge of any NBS program is to avoid false negatives while minimizing false positives and thereby reducing the number of newborns who require unnecessary clinical follow up. Typically, screening algorithms for disorders of propionate metabolism are based on C3, C3/C2 and other ratios as the primary marker for identifying a newborn that needs further clinical evaluation. Some programs repeated the first tier to confirm borderline positive screening results before referral or requesting another DBS specimen to repeat the NBS tests. This need for a second test prolongs the NBS process, resulting in delayed intervention that may lead to neurological impairment. In addition, using a single cut-off value for C3 and C3/C2 across all ages of sampling may misclassify a repeat measurement as normal [18]. Unlike first-tier screening, which typically uses direct flow injection, second-tier testing often employs LC-MS/MS to analyze additional disease-related metabolites, improving the specificity of NBS. MCA is unaffected by gestational age [12] and has been implemented in four countries as a second-tier marker. Although MCA concentrations are highly variable and affected by dietary protein intake and kidney function [19], MCA is generally accepted as a reliable marker for PA, although it can also be elevated (to a lesser extent) in patients with MMA [20]. The utilization of MCA avoids unnecessary recalls and improves PPV for PA. The Netherlands has also adopted this approach. However, the longer turnaround time for measuring MCA, which is performed only twice a week in one center in the Netherlands, can delay confirmation of the NBS positive result and thereby postpone timely referral and early intervention in affected newborns. Four of the European programs included a first-tier algorithm in which newborns with markedly elevated first-tier results are referred immediately for consultation and confirmatory testing, prior to the acquisition of second-tier test results.

We re-evaluated first-tier screening data from 11 confirmed cases of PA identified over a six-year period of screening for this disorder in the Netherlands. Several cut-offs and ratios were tested to assess their utility in enabling direct referral. Notably, our data show that immediate referral, without second-tier testing, for PA is warranted for samples with a high C3/C2 ratio (≥0.75). The implementation of this strategy would have resulted in earlier referral (a time gain of 2 to 6 days) of 10 out of the 11 PA confirmed cases. The single exception would have been referred following completion of second-tier analysis. An incidental but noteworthy finding was that one patient referred based on this proposed protocol was subsequently diagnosed with MMA, which is not problematic given that MMA is also included in the Dutch NBS panel and initial management of both disorders is the same, with the exception of providing hydroxocobalamin in MMA. This result formed the basis for the development of the analyte-based decision algorithm (Figure 1). For cases in which the C3/C2 ratio does not exceed 0.75, but for which milder abnormal biochemical patterns are determined in the first tier, a measurement of MCA, according to the current protocol, is still recommended. This can be performed within the standard response time as defined by current national NBS protocols. Our data show that it is possible to discriminate severely affected PA patients based on the first tier only, which will enable a more timely response to the initial episode of metabolic decompensation in early-onset PA patients. It should be noted that, due to differences between European NBS programs, this algorithm should be adapted to the respective national NBS organization structures and cut-off values.

Free carnitine levels can significantly influence both the formation and detection of acylcarnitines. In two of the detailed cases (Case 2 and 4), a second DBS was requested due to a low C0 concentration (3.4 µmol/L and 2.1 µmol/L), in line with the Dutch NBS protocol when primary carnitine uptake disorder (OMIM# 212140) is suspected. In both cases, the low C0 did not result in a more pronounced C3 concentration, as there was insufficient carnitine to bind the accumulating acyl groups, potentially leading to an underestimation of the metabolic disturbance. A normal C3 concentration, when seen alongside a low C0, may thus still be consistent with PA and warrants further investigation [21]. Similarly, a reported case of cobalamin C (CblC) disease that was missed during NBS presented with low C0, normal C3, and borderline C3/C2, further emphasizing the limitations of current markers, when used in isolation [22]. However, using the proposed cut-off of C3/C2 ≥ 0.75 µmol/L, patients 2 and 4 could have been referred immediately as suspected PA patients despite the low C0 in their NBS results.

PA, historically known as one of the ketotic hyperglycinemias, not only leads to acylcarnitine abnormalities, but also involves the accumulation of propionyl-CoA and related toxic metabolites that can inhibit the mitochondrial glycine cleavage system [23]. Although hyperglycinemia can support the diagnosis of PA in the right clinical and biochemical context, glycine concentrations may remain normal in the neonatal period despite the underlying metabolic defect (see also Supplementary Table S1). In the follow-up of Case 1, glycine concentrations were normal during the first months of life but consistently rose over the subsequent five months. Consequently, propionylglycine was absent in the organic acid profile during the period of normal glycine concentrations but became detectable during hyperglycinemia. The exact cause of this observation is not well understood. In neonates, the reabsorption of glycine may not be as efficient as in adults, resulting in a higher proportion of filtered glycine being excreted in the urine [24]. Propionyl-CoA can be conjugated to both glycine and carnitine, and the relative affinity for either conjugate may also be influenced by pH [25], with glycine conjugation favored during ketoacidosis. Furthermore, Anzmann et al. [26] suggested that increased synthesis from serine due to mitochondrial dysfunction/oxidative stress may also contribute to elevated plasma concentrations of glycine found in PA. Recently, it was discovered that widespread post-translational modification, specifically methylmalonylation, can inhibit enzymes in the glycine cleavage pathway in MMA [27]. From our survey, it became clear that glycine is not included as a marker for PA in the 13 European screening programs (which completed and returned the questionnaire) and is not regarded as informative in the CLIR database. Based on our data, we conclude that an elevated glycine concentration is not specific for PA in the context of NBS, and a normal glycine concentration does not rule out PA.

In conclusion, while enhancing the quality of NBS for PA through the implementation of second-tier strategies is essential for optimizing the PPV, this also leads to a diagnostic delay in early-onset patients. This may lengthen the duration of the initial metabolic decompensation and cause irreversible brain damage. Our results demonstrate that using a C3/C2 ratio of ≥0.75 for immediate referral can accelerate the diagnosis of early-onset PA patients, which may improve their outcome. Meanwhile, specimens with milder biochemical abnormalities in the first-tier could be temporarily held until second-tier testing is completed. Based on these considerations, we propose a revision of the current Dutch screening algorithm for PA which could be adopted in other countries as well.

## Figures and Tables

**Table 1:** Quantitative analysis of selected metabolic biomarkers in NBS DBS (in µmol/L) and their ratios in four early-onset PA samples compared to 99th percentile reference values.

Marker	Case 1		Case 2		Case 3		Case 4		
	Tier 1	Tier 2	Tier 1	Tier 2	Tier 1	Tier 2	Tier 1	Tier 2	99th percentile reference
C3	17.30		12.36		44.95		7.80		4.36
C0	18.47		3.38		129.7		2.10		43.7
C3/C0	0.93		3.65		0.35		3.71		0.23
C3/C2	1.23		2.75		5.31		1.35		0.23
C3/C16	3.01		7.58		40.90		5.95		1.89
MCA		25.05		44.10		28.90		29.54	

**Table 2:** The number of datasets that would be immediately referred versus those requiring second-tier testing based on potential markers/combination of markers, including: C3, C3/C0, C3 + C3/C0, C3/C2 and C3/C16 in the following categories: PA TP (true positive datasets for PA), PA FP (false positive datasets for PA), MMA TP (True positive datasets for MMA), MMA FP (false positive datasets for MMA) and negative (datasets classified as negative following second-tier testing).

		C3 ≥ 20	C3/C0 ≥ 0.5	C3 ≥ 20 or C3/C0 ≥ 0.5	C3/C2 ≥ 0.75	C3/C16 ≥ 2
PA TP (*n* = 11)	Direct referral (early onset)	7 (1)	6 (3)	10 (4)	10 (4)	10 (4)
	Second-tier (early onset)	4 (3)	5 (1)	1 (0)	1 (0)	1 (0)
PA FP (*n* = 5)	Direct referral	0	0	0	0	2
	Second-tier	5	5	5	5	3
MMA TP (*n* = 8)	Direct referral	1	3	3	1	5
	Second-tier	7	5	5	7	3
MMA FP (*n* = 105)	Direct referral	0	0	0	0	30
	Second-tier	105	105	105	105	75
Negative (*n* = 5943)	Direct referral	0	2	2	0	776
	Second-tier	5943	5941	5941	5943	5167

**Table 3:** NBS for PA: a comparison of the Dutch NBS program with other European NBS programs. # In 2002, screening for PA was implemented in the Tuscany region. * Second tier is currently being implemented.

		The Netherlands	Austria	Belgium (Flanders)	Belgium (Brussel/Wallonia)	Denmark	Finland	Hungary	Iceland	Italy	Norway	Poland	Slovak Republic	Slovenia	Sweden
Year of implementation		2019	2002	2007	2012-B 2014-W	2003	2015	2007	2008	2016 #	2012	2004	2013	2018	2010
Target Condition		LO	All	All	All	All	All	All	All	All	All	EO	EO	All	All
Age at heelprick (hours)		72–96	36–72	48–96	48–96	48–72	36–120	48–72	48–72	48–72	48–72	48–72	72–120	48–72	48–72
# of 1st Tier (×1000) annually			80	36	47	57	44	80	4.5	30	54	250	50	17–18	100
# of 2nd Tier (×10) annually			n.a.	0.5	35	n.a.	n.a. *	n.a.	n.a.	3–4	16.2	29	n.a.	n.a.	15–17
Expedited referral based on 1st Tier				●	●							●			●
1st Tier Primary Markers	C3	●	●	●	●	●	●	●	●	●	●	●	●	●	
	C17		●						●	●					
	C3/C2	●	●	●	●	●	●	●	●	●	●	●	●	●	●
	C3/C0		●					●		●					●
	C3/C16	●	●				●		●	●			●	●	●
	C4/C3										●				●
	C3/Met		●				●						●		●
	C14/C3				●										●
	(C16 + C18)/C3				●										●
	Met								●	●					●
2nd Tier Markers	Free 3-OH									●					
	Propionic acid				●					●					
	Propionyl Glycine									●					
	MMA	●		●	●					●	●	●			●
	MCA	●								●	●	●			●
	Homocysteine			●							●	●			●
	Methionine														●
	CLIR						●		●						●

## Data Availability

The datasets presented in this article are not readily available because the data are part of an ongoing study. Requests to access the datasets should be directed to the corresponding author.

## Supplementary Materials

The following supporting information can be downloaded at: https://www.mdpi.com/article/10.3390/ijns12010001/s1, Table S1: Glycine concentration in PA patients and the reference population.

## Abbreviations

The following abbreviations are used in this manuscript:

NBS	Newborn screening
PA	Propionic aciduria
MMA	Methylmalonic aciduria
PCC	Propionyl-CoA carboxylase
Propionyl-CoA	Propionyl-coenzyme A
C3	Propionylcarnitine
MCA	Methylcitric acid
MMA^mb^	Methylmalonic acid (MMA^mb^; mb refers to metabolite)
DBS	Dried blood spot
LC-MS/MS	Liquid chromatography tandem mass spectrometry
PPV	Positive predictive value
DOL	Day of life
PICU	Pediatric intensive care unit
C4DC	Methylmalonylcarnitine
CVVHDF	Continuous veno-venous hemodiafiltration
CLIR	Collaborative Laboratory Integrated Reports
CblC	Cobalamin C
