# Supplementary material for: Too Early to Tell? Balancing Diagnostic Accuracy of Newborn Screening for Propionic Acidemia Versus a Timely Referral
Source: Int J Neonatal Screen. 2025 Dec 24;12(1):1. doi: 10.3390/ijns12010001 (PMC12821464; doi:10.3390/ijns12010001)
Supplement: Supplementary file 1 [file IJNS-12-00001-s001.zip › IJNS-4034288-supplementary.pdf]

Supplementary information

Supplementary Table S1. Glycine concentration in PA patients and the reference population.

Classification	Glycine (µmol/L)
PA (1)	315.7
PA (2)	453.22
PA (3)	705.99
PA (4)	438.44
PA (5)	421.72
PA (6)	697.67
PA (7)	531.71
PA (8)	504.76
PA (9)	231.98
PA (10)	558.32
PA (11)	563.09
Reference 1st percentile	229.66
Reference $25t^{h}$ percentile	331.77
Reference 50th percentile	386.18
Reference 75th percentile	455.18
Reference 99th percentile	762.99
